# Supplementary material for: A systematic review on nomophobia prevalence: Surfacing results and standard guidelines for future research
Source: PLoS One. 2021 May 18;16(5):e0250509. doi: 10.1371/journal.pone.0250509 (PMC8130950; doi:10.1371/journal.pone.0250509)
Supplement: S1 Appendix — A: Studies included in the qualitative synthesis. B: A selection of studies not included in the qualitative synthesis. (PDF) [file pone.0250509.s001.pdf]

A systematic review on nomophobia prevalence: Surfacing results and standard guidelines for future research

Ana C. León-Mejía[1]◕*, Mónica Gutiérrez-Ortega[1]◕, Isabel Serrano-Pintado [2], Joaquín González-Cabrera[1]

1 Department of Psychology, Faculty of Education, Universidad Internacional de La Rioja (UNIR), Spain
2 Department of Psychology, Faculty of Psychology, Universidad de Salamanca, Spain

◕These authors contributed equally to this work.

* Corresponding author:
✉: aleon@unir.net

S1. Appendix A: Studies included in the qualitative synthesis

Authorship	Year	Journal of publication	Country
1. Abdulmohsin Suli Man [1]	2019	International Journal of Computer Science and Network Security	Saudi Arabia
2. Adawi et al. [2]	2018	JMIR mHealth and uHealth	Italy
3. Adawi et al. [3]	2019	Psychology Research and Behavior Management	Italy
4. Adnan and Gezgin [4]	2016	Journal of Faculty of Educational Sciences	Turkey
5. Aguilera-Manrique et al. [5]	2018	PLOS ONE	Spain
6. Ahmed et al. (2019a) [6]	2019	Journal of Public Health	India
7. Ahmed et al. (2019b) [7]	2019	Indian Journal of Psychiatry	India
8. Aini et al. [8]	2018	Jurnal Ners Widya Husada Semarang	Indonesia
9. Aktay and Hanife [9]	2019	International Technology and Education Journal	Turkey
10. Al-Balhan et al. [10]	2018	Psychology Research and Behavior Management	Kuwait
11. Ali et al. [11]	2017	Advanced Science Letters	Malasyia
12. Anushri et al. [12]	2018	Journal of Nursing Research	India
13. Apak and Yaman [13]	2019	Addicta: The Turkish Journal in Addictions	Turkey
14. Arpaci et al. [14]	2017	Information Development	Turkey
15. Arpaci et al. [15]	2017	Information Development	Turkey
16. Arpaci et al. [16]	2017	Journal of Medical Internet Research	Turkey
17. Asensio Chico et al. [17]	2018	Medicina de Familia. SEMERGEN	Spain
18. Ayar et al. [18]	2018	CIN: Computers, Informatics, Nursing	Turkey
19. Bartwal and Nath [19]	2019	Medical Journal Armed Forces	India
20. Batool and Ayesha [20]	2019	Peshawar Journal of Psychology and Behavioral Sciences	Turkey
21. Bernardini [21]	2018	Polygree	Italy
22. Bivin et al. [22]	2013	Reviews of Progress	India
23. Bragazzi et al. [23]	2019	JMIR Mental Health	Italy

24.	Bülbüloğlu et al. [24]	2019	Journal of Substance Use	Turkey
25.	Cain and Malcom [25]	2019	American Journal of Pharmaceutical Education	USA
26.	Chandak et al. [26]	2017	International Journal of Indian Psychology	India
27.	Chemara and Octaviani [27]	2017	Journal of Innovative Research in Social Sciences & Humanities	Indonesia
28.	Chukwuemeka et al. [28]	2017	Practicum Psychologia	Nigeria
29.	Daei et al. [29]	2019	International Journal of Preventive Medicine	Iran
30.	Darvishi et al. [30]	2019	Open Access Macedonian Journal of Medical Sciences	Iran
31.	Dasgupta et al. [31]	2017	Indian Journal of Public Health	India
32.	Datta et al. [32]	2016	Journal of Evolution of Medical and Dental Sciences	India
33.	Davie and Hilber [33]	2017	International Association for Development of the Information Society	Germany
34.	Deryakulu and Ursavaş [34]	2019	Addicta: The Turkish Journal on Addictions	Turkey
35.	Dixit et al. [35]	2010	Indian Journal of Community Medicine	India
36.	Dongre et al. [36]	2017	National Journal of Community Medicine	India
37.	Elyasi et al. [37]	2018	Addiction & Health	Iran
38.	Farooqui et al. [38]	2016	Journal of Mental Health	India
39.	Fitz et al. [39]	2019	Computers in Human Behavior	USA
40.	Galhardo et al. [40]	2020	Community Mental Health Journal	Portugal
41.	Gentina et al. [41]	2018	Computers & Education	USA
42.	Gezgin and Çakır [42]	2016	Journal of Human Sciences	Turkey
43.	Gezgin et al. (2017a) [43]	2017	Trakya University Journal of Education Faculty	Turkey
44.	Gezgin (2017b) [44]	2017	European Journal of Education Studies	Turkey
45.	Gezgin et al. (2018a) [45]	2018	International Journal of Research in Education and Science	Turkey
46.	Gezgin et al. (2018b) [46]	2018	International Journal of Research in Education and Science	Turkey
47.	Gezgin et al.(2018c) [47]	2018	Cypriot Journal of Educational Sciences	Turkey
48.	González-Cabrera et al. [133]	2018	Actas Españolas de Psiquiatría	Spain
49.	Gutiérrez-Puertas et al. [132]	2019	Nurse Education in Practice	Spain/Portugal

50.	Gutiérrez-Puertas et al. [48]	2016	CIN: Computers, Informatics, Nursing	Spain
51.	Han et al. [49]	2017	Cyberpsychology, Behavior, and Social Networking	Turkey
52.	Harish and Bharath [50]	2018	International Journal Of Community Medicine And Public Health	India
53.	Jianling and Chang [51]	2018	Current Psychology	China
54.	Jilisha et al. [52]	2019	Indian Journal of Psychological Medicine	India
55.	Jones et al. [53]	2019	Journal of Strength and Conditioning Research	Australia
56.	Kanmani [54]	2017	The International Journal of Indian Psychology	Turkey
57.	Kar [55]	2017	Journal of Bio Innovation	India
58.	Kara et al. [56]	2019	Journal Behaviour & Information Technology	Turkey
59.	Kaur et al. [57]	2015	International Journal of Psychiatric Nursing	India
60.	King et al [?]	2014	Clinical Practice and Epidemiology in Mental Health	Brasil
61.	King et al [58]	2017	Journal of Addiction Research &Therapy	Brasil
62.	Koay et al. [59]	2019	Southeast Asia Psychology Journal	Malasya
63.	Lee et al. [60]	2012	Journal of Physical Therapy Science	Korea
64.	Lee et al. [61]	2017	Applied Cognitive Psychology	USA
65.	Lee et al. [62]	2018	Heliyon	USA
66.	Lin et al. [63]	2018	Journal of Behavioral Addiction	Iran
67.	Louragli et al. [64]	2018	Problems of Psychology in the 21st Century	Morocco
68.	Mallya et al. [65]	2018	National Journal of Physiology, Pharmacy and Pharmacology	India
69.	Mane [66]	2017	Imperial Journal of Interdisciplinary Research	India
70.	Matoza-Báez and Carballo-Ramírez [67]	2016	Ciencia e Investigación Médica Estudiantil Latinoamericana	Paraguay
71.	Mendoza et al. [68]	2018	Computers in Human Behavior	USA
72.	Menezes and Pangam [69]	2017	International Journal of Psychiatric Nursing	India
73.	Peris et al. [70]	2018	Revista de Psicología Clínica con Niños y Adolescentes	Spain
74.	Muralidhar et al. [71]	2017	International Journal of Medical Science and Public Health	India
75.	Musa et al. [72]	2017	Advanced Science Letters	Malasyia

76.	Nagpal and Ramanpreet [73]	2016	Indian Journal of Health & Wellbeing	India
77.	Nawaz et al. [74]	2017	Journal of Technology in Behavioral Sciences	Pakistan
78.	Nezih [75]	2018	International Journal of Research in Education and Science	Turkey
79.	Nidhin et al. [76]	2014	Asian Journal of Nursing Education and Research	India
80.	Nisreen et al. [77]	2018	The Medical Journal of Tikrit University	Irak
81.	Olivencia-Carrión et al. [78]	2018	Psychiatry Research	Spain
82.	Ozdemir et al. [79]	2018	Eurasia Journal of Mathematics, Science and Technology Education	Pakistan/Turkey
83.	Pavithra et al. [80]	2015	National Journal of Community Medicine	India
84.	Prasad et al. [81]	2017	Journal of Clinical and Diagnostic Research	India
85.	Priyanka and Kishanth [82]	2016	International Journal of Nursing Education	India
86.	Ramos-Soler et al. [130]	2017	Salud y Drogas	Spain
87.	Rangka et al. [83]	2018	Journal of Physics: Conference Series	Indonesia
88.	Rosales-Huamani et al. [84]	2019	Applied Sciences	Peru
89.	Sakiroglu et al. [85]	2017	International Journal of Psycho-Educational Sciences	Turkey
90.	Salwa [86]	2017	Journal of Research in Curriculum Instruction and Educational Technology	Saudi Arabia
91.	Sebin and Jinesh [87]	2018	International Journal of Engineering Technology Science and Research	India
92.	Semerci [88]	2019	Bartin University Journal of Faculty of Education	Turkey
93.	Sethia et al. [89]	2018	International Journal of Community Medicine and Public Health	India
94.	Sharma et al. [90]	2015	International Journal of Research in Medical Sciences	India
95.	Silva Giraldo et al. [91]	2018	Boletín Redipe	Colombia
96.	Tams et al. [92]	2018	Computers in Human Behavior	Canada
97.	Tavolacci et al. [93]	2015	European Journal of Public Health	France
98.	Torres-Salazar et al. [94]	2018	Revista Internacional de Investigación en Adicciones	Mexico
99.	Uysal et al. [95]	2016	The Global eLearning Journal	India
100.	Veerapu et al. [96]	2019	International Journal of Community Medicine And Public Health	India
101.	Yasan Ak and Yildirim [97]	2018	International Journal on New Trends in Education and their Implications	Turkey

102. Yavuz et al. [98]	2014	The Turkish Journal of Pediatrics	Turkey
103. Yildirim [126]	2014	Iowa State University	USA
104. Yildirim and Correia (2015a) [127]	2015	Computers in Human Behavior	Turkey
105. Yildirim and Correia (2015b) [128]	2015	Learning and Collaboration Technologies	USA
106. Yildirim et al. (2015) [129]	2015	Information Development	USA
107. Yildiz-Durak [99]	2018	Addicta: The Turkish Journal on Addictions	Turkey
108. Yildiz-Durak [100]	2019	The Social Science Journal	Turkey

S1. Appendix B: A selection of studies not included in the qualitative synthesis

The literature on NMP is huge and there is a very large number of studies worth reading that we did not include in the qualitative synthesis due to our research questions and inclusion criteria. In the following list there is a selection of some of them that we find particularly interesting because of the perspective followed: focus on qualitative research, clinical view and therapeutical approach, and theoretical discussion of nomophobia from a novel point of view.

Reference of the study	Year	Journal of publication	Country
King et al. [125]	2010	Cognitive and Behavioral Neurology	Brasil
King et al. [101]	2013	Computers in Human Behavior	Brasil
Bragazzi and Del Puente [102]	2014	Psychology Research and Behavior Management	Italy
Cheever et al. [103]	2014	Computers in Human Behavior	US
Tran [104]	2016	UC Merced Undergraduate Research Journal	US
Rosen et al. [105]	2016	Sleep Health in Early Care and Education	US
Pistilli and Cain [106]	2016	Currents in Pharmacy Teaching and Learning	US
Pathak [107]	2016	Journal of Research in Humanities & Soc. Sciences	India
Belk [108]	2016	Current Opinion in Psychology	Canada
Aagaard [109]	2016	AI & Society	Denmark
Kuss and Griffiths [110]	2017	International Journal of Environmental Research and Public Health	UK
García-Umaña [111]	2017	Dilemas Contemporáneos	Ecuador
Prasyatiani et al. [112]	2017	4th International Conference on Education and Social Sciences	Indonesia
Papaconstantinou et al. [113]	2017	Sleep Medicine	US
Akun and Andreani [114]	2017	International Conference on Human System Interactions	Indonesia
van Velthoven et al. [115]	2018	Digital Health	UK
Güzel [116]	2018	Journal of Academic Perspective on Social Studies	Saudi Arabia
Wang and Suh [117]	2018	The 2018 CHI Conference	Hong Kong
Bychkov and Young [118]	2018	Big Data in Engineering Applications	US

Bhattacharya et al. [119]	2018	Journal of Family Medicine and Primary Care	India
Liu et al. [120]	2019	International Journal of Environmental Research and Public Health	China, Canada and US
Gezgin et al. [121]	2019	Bartın University Journal of Faculty of Education	Turkey
Anshari et al. [122]	2019	Vulnerable Children and Youth Studies	Brunei
Betoncu and Ozdamli [123]	2019	TEM Journal	Turkey
Park and Kaye [124]	2019	Mobile Media & Communication	US
Wahyuningtyas et al. [131]	2020	Jurnal Bimbingan Konseling	Indonesia
León-Mejía et al [134]	2020	Adicciones	Spain

References

1. Abdulmohsin Suli Man A. An Empirical Study of Nomophobia Behavior of Saudi Learners. International Journal of Computer Science and Network Security. 2019;19(10):20–25.

2. Adawi M, Bragazzi NL, Argumosa-Villar L, Boada-Grau J, Vigil-Colet A, Yildirim C, et al. Translation and Validation of the Nomophobia Questionnaire (NMP-Q) in the Italian Language: Insights From Factor Analysis. JMIR mHealth and uHealth. 2018;6. doi:10.2196/mhealth.9186.

3. Adawi M, Zerbetto R, Simona Re T, Bisharat B, Mahamid M, Amital H, et al. Psychometric Properties of the Brief Symptom Inventory in Nomophobic Subjects: Insights from Preliminary Confirmatory Factor, Exploratory Factor, and Clustering Analyses in a Sample of Healthy Italian Volunteers. Psychology Research and Behavior Management. 2019;12:145–154. doi:10.2147/PRBM.S173282.

4. Adnan M, Gezgin D. A Modern Phobia: Prevalence of Nomophobia Among College Students. Journal of Faculty of Educational Sciences. 2016;49:2016–2141. doi:10.1501/Egifak_0000001378.

5. Aguilera-Manrique G, Márquez-Hernández VV, Alcaraz-Córdoba T, Granados-Gámez G, Gutiérrez-Puertas V, Gutiérrez-Puertas L. The Relationship Between Nomophobia and the Distraction Associated with Smartphone Use Among Nursing Students in their Clinical Practicum. PLOS ONE. 2018;13(8):e0202953.

6. Ahmed S, Pokhrel N, Roy S, Samuel A. Impact of Nomophobia: A Nondrug Addiction Among Students of Physiotherapy Course Using an Online Cross-Sectional Survey. Indian Journal of Psychiatry. 2019;61:77–80. doi:10.4103/psychiatry.IndianJPsychiatry_361_18.

7. Ahmed S, Akter R, Pokhrel N, Samuel A. Prevalence of Text Neck Syndrome and SMS Thumb Among Smartphone Users in College-Going Students: A Cross-Sectional Survey Study. Journal of Public Health. 2019; p. 1–6. doi:10.1007/s10389-019-01139-4.

8. Aini K, Retnaningsih D, ATrisnaja PA. The Influence of Thought Stopping Therapy to Nomophobia on Nursing College Students of Widya Husada Semarang. Jurnal Ners Widya Husada Semarang. 2018;3(3).

9. Aktay EG, Hanife PK. Primary School Teacher Candidates and Nomophobia. International Technology and Education Journal. 2019;3(1):16–24.

10. Al-Balhan E, KHabbache H, Watfa A, Simona Re T, Zerbetto R, Bragazzi NL. Psychometric Evaluation of the Arabic Version of the Nomophobia Questionnaire: Confirmatory and Exploratory Factor Analysis. Implications From a Pilot Study in Kuwait Among University Students. Psychology Research and Behavior Management. 2018;11. doi:10.2147/PRBM.S169918.

11. Ali A, Muda M, Ridzuan AR, Nuji MNN, Izzamuddin M, Imma D. The Relationship Between Phone Usage Factors and Nomophobia. Advanced Science Letters. 2017;23:7610–7613. doi:10.1166/asl.2017.9534.

12. Anushri C, Darshana T, B M, Pranali W, Sneha Y, Lakshmanan M. A Study to Assess Nomophobia among Higher Secondary Students in Order to Develop a Pamphlet Regarding Prevention of Nomophobia in Nagpur City. Journal of Nursing Research. 2018;3(1):53–57.

13. Apak E, Yaman OM. The Prevalence of Nomophobia among University Students and Nomophobia's Relationship with Social Phobia: The Case of Bing'Ol University. Addicta: The Turkish Journal on Addictions. 2019;6(3):611–629. doi:10.15805/addicta.2019.6.3.0078.

14. Arpaci I, Baloğlu M, Kesici S. A Multi-Group Analysis of the Effects of Individual Differences in Mindfulness on Nomophobia. Information Development. 2017;35. doi:10.1177/0266666917745350.

15. Arpaci I. Culture and Nomophobia: The Role of Vertical Versus Horizontal Collectivism in Predicting Nomophobia. Information Development. 2017;35:026666691773011. doi:10.1177/0266666917730119.

16. Arpaci I, Baloğlu M, Özteke Kozan H, Ş K. Individual Differences in the Relationship Between Attachment and Nomophobia Among College Students: The Mediating Role of Mindfulness. Journal of Medical Internet Research. 2017;19(2):e404. doi:10.2196/jmir.8847.

17. Asensio Chico I, Díaz Maldonado L, Garrote Moreno L. Nomofobia: Enfermedades del Siglo Veintiuno. Medicina de Familia. 2018;44(7):e117–e118. doi:10.1016/j.semerg.2018.05.002.

18. Ayar D, Özalp Gerçeker G, Özdemir EZ, Bektas M. The Effect of Problematic Internet Use, Social Appearance Anxiety, and Social Media Use on Nursing Students' Nomophobia Levels. CIN: Computers, Informatics, Nursing. 2018;36(12).

19. Bartwal J, Nath B. Evaluation of Nomophobia Among Medical Students Using Smartphone in North India. Medical Journal Armed Forces India. 2019;doi:10.1016/j.mjafi.2019.03.001.

20. Batool I, Ayesha Z. Nomophobia an Emerging Fear: An Experimental Exploration among University Students. Peshawar Journal of Psychology and Behavioral Sciences. 2019;5(1).

21. Bernardini J. Nomophobia and Digital Natives. An Empirical Research Study Among Young Italians. Polygree. 2018;15.

22. Bivin JB, Preeti M, Praveen C, Jinto P. Nomophobia - Do We Really Need to Worry About? A Cross Sectional Study on Nomophobia Severity Among Male Under Graduate Students of Health Sciences. Reviews of Progress. 2013;1(1):1–5.

23. Bragazzi NL, Simona Re T, Zerbetto R. The Relationship Between Nomophobia and Maladaptive Coping Styles in a Sample

of Italian Young Adults: Insights and Implications From a Cross-Sectional Study. JMIR Mental Health. 2019;6(4):e13154–e13154. doi:10.2196/13154.

24. Bülbüloğlu S, Özdemir A, Kapıkıran G, Sarıtaş S. The Effect of Nomophobic Behavior of Nurses Working at Surgical Clinics on Time Management and Psychological Well-Being. Journal of Substance Use. 2019; p. 1–6. doi:10.1080/14659891.2019.1692926.

25. Cain J, Malcom D. An Assessment of Pharmacy Students' Psychological Attachment to Smartphones at Two Colleges of Pharmacy. American Journal of Pharmaceutical Education. 2019;83:7136. doi:10.5688/ajpe7136.

26. Chandak P, Singh D, Faye A, Gawande S, Tadke R, Kirpekar V, et al. An Exploratory Study of Nomophobia in Post Graduate Residents of a Teaching Hospital in Central India. International Journal of Indian Psychology. 2017;4(3). doi:10.25215/0403.147.

27. Chemara Z, Octaviani UF. Nomophobia Around Us! Journal of Innovative Research in Social Sciences & Humanities. 2017;1(1):1–35.

28. Chukwuemeka FO, Obi-Nwosu H, Obikwelu VC. Nomophobia Among Undergraduate: Predictive Influence of Personality Traits. Practicum Psychologia. 2017;7(2):64–74.

29. Daei A, Ashrafi-rizi H, Soleymani MR. Nomophobia and Health Hazards: Smartphone Use and Addiction Among University Students. International Journal of Preventive Medicine. 2019;10:202. doi:10.4103/ijpvm.IJPVM_184_19.

30. Darvishi M, Noori M, Nazer MR, Sheikholeslami S, Karimi E. Investigating Different Dimensions of Nomophobia among Medical Students: A Cross-Sectional Study. Open Access Macedonian Journal of Medical Sciences. 2019;7(4):573–578. doi:10.3889/oamjms.2019.138.

31. Dasgupta P, Bhattacherjee S, Dasgupta S, Roy J, Mukherjee A, Biswas R. Nomophobic Behaviors Among Smartphone Using Medical and Engineering students in Two Colleges of West Bengal. Indian Journal of Public Health. 2017;61:199–204. doi:10.4103/ijph.IJPH_81_16.

32. Datta S, Nelson V, Simon S. Mobile Phone Use Pattern and Self Reported Health Problems Among Medical Students. Journal of Evolution of Medical and Dental Sciences. 2016;5. doi:10.14260/jemds/2016/259.

33. Davie N, Hilber T. Nomophobia: Is Smartphone Addiction a Genuine Risk for Mobile Learning? International Association for Development of the Information Society. 2017;.

34. Deryakulu D, Ursavaş Ö. Genetic and Environmental Sources of Nomophobia: A Small-Scale Turkish Twin Study. Addicta: The Turkish Journal on Addictions. 2019;6. doi:10.15805/addicta.2019.6.1.0028.

35. Dixit S, Shukla H, Bhagwat A, Bindal A, Goyal A, Zaidi A, et al. A Study to Evaluate Mobile Phone Dependence Among Students of a Medical College and Associated Hospital of Central India. Indian Journal of Community Medicine. 2010;35(2):339–341.

36. Dongre AS, Inamdar IF, Gattani PL. Nomophobia: A Study to Evaluate Mobile Phone Dependence and Impact of Cell Phone on Health. National Journal of Community Medicine. 2017;8(11):688–693.

37. Elyasi F, Hakimi B, Islami-Parkoohi P. The Validity and Reliability of the Persian Version of Nomophobia Questionnaire. Addiction & Health. 2018;10:231–241. doi:10.22122/ahj.v10i4.647.

38. Farooqui IA, Pore P, Gothankar J. Nomophobia: An Emerging Issue in Medical Institutions? Journal of Mental Health. 2016;27(5):438–41. doi:10.13140/RG.2.2.34355.12328.

39. Fitz N, Kushlev K, Jagannathan R, Lewis T, Paliwal D, Ariely D. Batching Smartphone Notifications Can Improve Well-Being. Computers in Human Behavior. 2019;101:84–94. doi:10.1016/j.chb.2019.07.016.

40. Galhardo A, Loureiro D, Raimundo E, Massano-Cardoso I, Cunha M. Assessing Nomophobia: Validation Study of the European Portuguese Version of the Nomophobia Questionnaire. Community Mental Health Journal. 2020;doi:10.1007/s10597-020-00600-z.

41. Gentina E, Tang TLP, Dancoine PF. Does Gen Z's Emotional Intelligence Promote iCheating (Cheating with iPhone) Yet Curb iCheating Through Reduced Nomophobia? Computers & Education. 2018;126:231–247. doi:10.1016/j.compedu.2018.07.011.

42. Gezgin DM, Çakır Ö. Analysis of Nomophobic Behaviors of Adolescents Regarding Various Factors. Journal of Human Sciences. 2016;13(2 SE):2504–2519.

43. Gezgin D, Sumuer E, Arslan O, Yildirim S. Nomophobia Prevalence Among Pre-service Teachers: A case of Trakya University. Trakya University Journal of Education Faculty. 2017;7.

44. Gezgin D. Exploring The Influence of The Patterns of Mobile Internet Use on University Students' Nomophobia Levels. European Journal of Education Studies. 2017;3(6):29–53. doi:10.5281/zenodo.572344.

45. Gezgin D, Çakır Ö, Yildirim S. The Relationship between Levels of Nomophobia Prevalence and Internet Addiction among High School Students: the factors influencing Nomophobia. International Journal of Research in Education and Science. 2018;4(1):215–225. doi:10.21890/ijres.383153.

46. Gezgin D, Hamutoglu N, Sezen-Gultekin G, Ayas T. The Relationship Between Nomophobia and Loneliness Among Turkish Adolescents. International Journal of Research in Education and Science. 2018;4(2):358–374.

47. Gezgin D, Hamutoglu N, Sezen-Gultekin G, Gemikonakli O. Relationship Between Nomophobia and Fear of Missing Out Among Turkish University Students. Cypriot Journal of Educational Sciences. 2018;13(4):549–561. doi:10.18844/cjes.v13i4.3464.

48. Gutiérrez-Puertas L, Hernández VV, Aguilera-Manrique G. Adaptation and Validation of the Spanish Version of the Nomophobia Questionnaire in Nursing Studies. CIN: Computers, Informatics, Nursing. 2016;34(10):470–475. doi:10.1097/CIN.0000000000000268.

49. Han S, Kim KJ, Kim JH. Understanding Nomophobia: Structural Equation Modeling and Semantic Network Analysis of Smartphone Separation Anxiety. Cyberpsychology, Behavior, and Social Networking. 2017;20(7):419–427. doi:10.1089/cyber.2017.0113.

50. Harish BR, Bharath J. Prevalence of Nomophobia Among the Undergraduate Medical Students of Mandya Institute of Medical Sciences, Mandya. International Journal Of Community Medicine And Public Health. 2018;5(12). doi:10.18203/2394-6040.ijcmph20184833.

51. Jianling M, Chang L. Evaluation of the Factor Structure of the Chinese Version of the Nomophobia Questionnaire. Current Psychology. 2018; p. 1–7. doi:10.1007/s12144-018-0071-9.

52. Jilisha G, J V, Menon V, Olickal J. Nomophobia: A Mixed-Methods Study on Prevalence, Associated Factors, and Perception Among College Students in Puducherry, India. Indian Journal of Psychological Medicine. 2019;41(6):541–548. doi:10.4103/IJPSYM.IJPSYM_130_19.

53. Jones M, Dawson B, Eastwood P, Halson S, Miller J, Murray K, et al. Influence of Electronic Devices on Sleep and Cognitive Performance During Athlete Training Camps. Journal of Strength and Conditioning Research. 2019;6. doi:10.1519/JSC.0000000000002991.

54. Kanmani A. Nomophobia-An Insight into Its Psychological Aspects in India. The International Journal of Indian Psychology. 2017;4(2):6–15.

55. Kar S. Prevalence of Nomophobia Among Medical Students in a Private College of Bhubaneswar, Odisha. Journal of Bio Innovation. 2017;6:914–920.

56. Kara M, Baytemir K, İnceman-Kara F. Duration of Daily Smartphone Usage as an Antecedent of Nomophobia: Exploring Multiple Mediation of Loneliness and Anxiety. Journal Behaviour & Information Technology. 2019;1(4).

57. Kaur A, Sharma P, Manu. A Descriptive Study to Assess the Risk of Developing Nomophobia among Students of Selected Nursing Colleges Ludhiana, Punjab. International Journal of Psychiatric Nursing. 2015;1:1. doi:10.5958/2395-180X.2015.00051.1.

58. King ALS, Guedes E, Simões Neto JP, Leite F, Nardi A. Nomophobia: Clinical and Demographic Profile of Social Network Excessive Users. Journal of Addiction Research & Therapy. 2017;08(4):1. doi:10.4172/2155-6105.1000339.

59. Koay, Yin K, Yahaya A, Sangryeol C, Maakip I, Voo P, et al. Smartphone Usage, Smartphone Addition, Internet Addition and Nomophobia University Malasya Sabah (UMS). Southeast Asia Psychology Journal. 2019;7:1–12.

60. Lee YS, Yang HS, Jeong CJ, Yoo YD, Jeong GY, Moon JS, et al. Changes in the Thickness of Median Nerves Due to Excessive Use of Smartphones. Journal of Physical Therapy Science. 2012;24:1259–1262. doi:10.1589/jpts.24.1259.

61. Lee S, Kim M, Mcdonough I, Mendoza J, Kim M. The Effects of Cell Phone Use and Emotion-Regulation Style on College Students' Learning. Applied Cognitive Psychology. 2017;31(3):360–366. doi:10.1002/acp.3323.

62. Lee S, Kim M, Mendoza JS, McDonough IM. Addicted to Cellphones: Exploring the Psychometric Properties Between the Nomophobia Questionnaire and Obsessiveness in College Students. Heliyon. 2018;4(11):e00895. doi:https://doi.org/10.1016/j.heliyon.2018.e00895.

63. Lin CY, Griffiths MD, Pakpour AH. Psychometric Evaluation of Persian Nomophobia Questionnaire: Differential Item Functioning and Measurement Invariance Across Gender. Journal of Behavioral Addiction. 1;7(100-108). doi:10.1556/2006.7.2018.11.

64. Louragli I, Ahami A, Khadmaoui A, Mammad K, Chaker Lamrani A. Evaluation of the Nomophobia's Prevalence and its Impact on School Performance Among Adolescents in Morocco. Problems of Psychology in the 21st Century. 2018;12:84–94. doi:10.33225/ppc/18.12.84.

65. Mallya NV, Sunil Kumar DR, Mashal S. A Study to Evaluate the Behavioral Dimensions of "Nomophobia" and Attitude Toward Smartphone Usage Among Medical Students in Bengaluru. National Journal of Physiology, Pharmacy and Pharmacology. 2018;8(11):1553–1557. doi:10.5455/njppp.2018.8.0827603092018.

66. Mane S. A Descriptive Study to Assess the Risk of Developing Nomophobia Among Students of Selected Nursing Colleges of Navi Mumbai. Imperial Journal of Interdisciplinary Research. 2017;3(8).

67. Matoza-Báez CM, Carballo-Ramírez MS. Nomophobia Levels on Medical Students from Paraguay, Year 2015. Ciencia e Investigación Médica Estudiantil Latinoamericana. 2016;21(1):28–30.

68. Mendoza JS, Pody BC, Lee S, Kim M, McDonough IM. The Effect of Cellphones on Attention and Learning: The Influences of Time, Distraction, and Nomophobia. Computers in Human Behavior. 2018;86:52–60. doi:10.1016/j.chb.2018.04.027.

69. Menezes PM, Pangam S. Prevalence, Awareness and Effects of Nomophobia Among Adolescents. International Journal of Psychiatric Nursing. 2017;3(2):16–18. doi:10.5958/2395-180X.2017.00015.9.

70. Peris M, Maganto C, Garaigordobil M. Escala de Riesgo de Adicción Adolescente a las Redes Sociales e Internet: Fiabilidad y Validez (ERA-RSI). Revista de Psicología Clínica con Niños y Adolescentes. 2018;5(2):30–36. doi:10.21134/rpcna.2018.05.2.4.

71. Muralidhar M, Sudarshan B, T V S, Gopi A, Fernandes S. Nomophobia and its Determinants Among the Students of a Medical College in Kerala. International Journal of Medical Science and Public Health. 2017;6:1046–1049. doi:10.5455/ijmsph.2017.0203115022017.

72. Musa R, Janiffa S, Rahman S. Who's at Risk for Smartphone Nomophobia and Pathology; The Young or Matured Urban Millennials? Advanced Science Letters. 2017;23:7486–7489. doi:10.1166/asl.2017.9504.

73. Nagpal SS, Ramanpreet K. Nomophobia: The Problem Lies at our Fingertips. Indian Journal of Health & Wellbeing. 2016;7(12):1135–1139.

74. Nawaz I, Sultana I, Muhammad JA, Shaheen A. Measuring the Enormity of Nomophobia Among Youth in Pakistan. Journal of Technology in Behavioral Sciences. 2017;2(3-4):149–155. doi:10.1007/s41347-017-0028-0.

75. Nezih O. Metaphoric Perceptions of High School Students about Nomophobia. International Journal of Research in Education and Science. 2018;5(2):437–449.

76. Nidhin A, Mathias J, Williams S. A Study to Assess the Knowledge and Effect of Nomophobia Among Students of Selected Degree Colleges in Mysore. Asian Journal of Nursing Education and Research. 2014;4(4):421–428.

77. Nisreen MI, Assala FM, Al-Maha YA. Assessment the Nomophobia Among Medical Students in Tikrit University College of Medicine. The Medical Journal of Tikrit University. 2018;24(2):78–94. doi:10.25130/mjotu.24.02.08.

78. Olivencia-Carrión M, Ferri-García R, Rueda M, Jiménez-Torres M, López-Torrecillas F. Temperament and Characteristics Related to Nomophobia. Psychiatry Research. 2018;266:5–10. doi:10.1016/j.psychres.2018.04.056.

79. Ozdemir B, Çakır Ö, Hussain I. Prevalence of Nomophobia Among University Students: A Comparative Study of Pakistani and Turkish Undergraduate Students. Eurasia Journal of Mathematics, Science and Technology Education. 2018;14:1519–1532. doi:10.29333/ejmste/84839.

80. Pavithra M, Madhukumar S, Mahadeva TM. A Study on Nomophobia. Mobile Phone Dependence Among Students of a Medical

College in Bangalore. National Journal of Community Medicine. 2015;6(2):340–344.

81. Prasad M, Basavaraj P, Singla A, Gupta R, Saha S, Kumar J, et al. Nomophobia: A Cross-sectional Study to Assess Mobile Phone Usage Among Dental Students. Journal of Clinical and Diagnostic Research. 2017;11(2):ZC34–ZC39. doi:10.7860/JCDR/2017/20858.9341.

82. Priyanka T, Kishanth O. A Quasi Experimental Study to Assess the Effect of Structured Teaching Programme on Knowledge Regarding Nomophobia among Students of Selected Colleges in District Jalandhar, Punjab. International Journal of Nursing Education. 2016;8(2):119–121. doi:10.5958/0974-9357.2016.00060.X.

83. Rangka I, Prasetyaningtyas W, Ifdil I, Ardi Z, Suranata K, Winingsih E, et al. Measuring Psychometric Properties of the Indonesian Version of the NoMoPhobia Questionnaire (NMPQ): Insight from Rasch Measurement Tool. Journal of Physics: Conference Series. 2018;1114(1):12127. doi:10.1088/1742-6596/1114/1/012127.

84. Rosales-Huamani J, Castillo-Sequera J, Guzman-Lopez R, Aroni-Vilca E, Matos C. Determining Symptomatic Factors of Nomophobia in Peruvian Students from the National University of Engineering. Applied Sciences. 2019;9:1814. doi:10.3390/app9091814.

85. Sakiroglu M, Gülada G, Uğurcan S, Kara N, Gandur T. The Mediator Effect of Mindfulness Awareness on The Relationship Between Nomophobia and Academic University Adjustment Levels in College Students. International Journal of Psycho-Educational Sciences. 2017;6(3):69–79.

86. Salwa AK. The Prevalence and Psychological Symptoms of Nomophobia among University Students. Journal of Research in Curriculum Instruction and Educational Technology. 2017;3(3):155–182. doi:10.12816/0042091.

87. Sebin S, Jinesh N. A Study on Impact of Smart Phone Usage on Health of College Going Students. International Journal of Engineering Technology Science and Research. 2018;5(3):684–689.

88. Semerci A. Nomophobia as the Predictor of Secondary School Students' Smartphone Addiction. Bartın Üniversitesi Eğitim Fakültesi Dergisi. 2019;8(3):947–965. doi:10.14686/BUEFAD.592443.

89. Sethia S, Melwani V, Melwani S, Priya A, Gupta M, Amreen K. A Study to Assess the Degree of Nomophobia Among the Undergraduate Students of a Medical College in Bhopal. International Journal of Community Medicine and Public Health. 2018;5(6):2442–2445.

90. Sharma N, Sharma P, Sharma N, Wavare R. Rising Concern of Nomophobia Amongst Indian Medical Students. International Journal of Research in Medical Sciences. 2015;3(3):705–707. doi:10.5455/2320-6012.ijrms20150333.

91. Silva Giraldo C, Almeida Salinas O, Corzo J, Rovira K, Suescun E. Identificación de la Relación Existente entre los Factores Predisponentes y Mantenedores en la Presencia de Nomofobia en los Estudiantes de PsicologÍa Vinculados a la Corporación Universitaria Minuto de Dios-Bucaramanga. Boletín Redipe. 2018;7(10):216–234.

92. Tams S, Legoux R, Léger PM. Smartphone Withdrawal Creates Stress: A Moderated Mediation Model of Nomophobia, Social Threat, and Phone Withdrawal Context. Computers in Human Behavior. 2018;81:1–9. doi:10.1016/j.chb.2017.11.026.

93. Tavolacci MP, Meyrignac G, Richard L, Déchelotte P, Ladner J. Problematic Use of Mobile Phone and Nomophobia Among French College Students: Marie-Pierre Tavolacci. European Journal of Public Health. 2015;25(3):206. doi:10.1093/eurpub/ckv172.088.

94. Torres-Salazar Q, Ramírez-Gurrola A, Castañón-Alvarado M, Aroña-Campos A, Betancourt-Araujo A, Carrillo-Cisneros M, et al. Asociación entre Nomofobia e Indicadores de Inteligencia en Estudiantes de Educación Superior. Revista Internacional de Investigación en Adicciones. 2018;4:19–24. doi:10.28931/riiad.2018.2.03.

95. Uysal Ş, Doç Y, Özen H, Canan M, Salih Z, Anadolu L, et al. Social Phobia in Higher Education: The Influence of Nomophobia on Social Phobia. The Global eLearning Journal. 2016;5(2):2–8.

96. Veerapu N, Philip R, Vasireddy H, Gurrala S, Kanna S. A Study on Nomophobia and its Correlation with Sleeping Difficulty and Anxiety Among Medical Students in a Medical College, Telangana. International Journal Of Community Medicine And Public Health. 2019;6(5):2074. doi:10.18203/2394-6040.ijcmph20191821.

97. Yasan Ak N, Yildirim S. Nomophobia Among Undergraduate Students: The Case of a Turkish State University. International Journal on New Trends in Education and Their Implications. 2018;9(4):11–20.

98. Yavuz M, Altan B, Bayrak B, Gündüz M, Bolat N. The Relationships Between Nomophobia, Alexithymia and Metacognitive Problems in an Adolescent Population. The Turkish Journal of Pediatrics. 2019;61. doi:10.24953/turkjped.2019.03.005.

99. Yildiz-Durak H. What Would You Do Without Your Smartphone? Adolescents' Social Media Usage, Locus of Control, and Loneliness as a Predictor of Nomophobia. Addicta: The Turkish Journal on Addictions. 2018;5(3):543–557. doi:10.15805/addicta.2018.5.2.0025.

100. Yildiz-Durak H. Investigation of Nomophobia and Smartphone Addiction Predictors Among Adolescents in Turkey: Demographic Variables and Academic Performance. The Social Science Journal. 2019;56(4):492–517. doi:10.1016/j.soscij.2018.09.003.

101. King ALS, Valença AM, da Silva AC, , Baczynski TP, de Carvalho MR, et al. Nomophobia: Dependency on virtual environments or social phobia? Computers in Human Behavior. 2013;29(1):140–144. doi:10.1016/j.chb.2012.07.025.

102. Bragazzi N, Del Puente G. A Proposal for Including Nomophobia in the New DSM-V. Psychology Research and Behavior Management. 2014;7:155–60. doi:10.2147/PRBM.S41386.

103. Cheever NA, Rosen LD, Carrier LM, Chavez A. Out of Sight is Not Out of Mind: The Impact of Restricting Wireless Mobile Device Use on Anxiety Levels among Low, Moderate and High Users. Computers in Human Behavior. 2014;37:290–297. doi:10.1016/j.chb.2014.05.002.

104. Tran D. Classifying Nomophobia as Smart-Phone Addiction Disorder. UC Merced Undergraduate Research Journal. 2016;9(1).

105. Rosen L, Carrier LM, Miller A, Rokkum J, Ruiz A. Sleeping with Technology: Cognitive, Affective, and Technology Usage Predictors of Sleep Problems Among College Students. Sleep Health in Early Care and Education. 2016;2(1):49–56. doi:10.1016/j.sleh.2015.11.003.

106. Pistilli N, Cain J. Using a Health Care Practice Framework to Address Smartphone Use in the Classroom. Currents in Pharmacy Teaching and Learning. 2016;8(2):247–253. doi:10.1016/j.cptl.2015.12.020.

107. Pathak NK. Digital Detox in India. Journal of Research in Humanities & Soc Sciences. 2016;4(8).

108. Belk R. Extended Self and the Digital World. Current Opinion in Psychology. 2016;10:50–54. doi:10.1016/j.copsyc.2015.11.003.

109. Aagaard J. Mobile devices, Interaction, and Distraction: A Qualitative Exploration of Absent Presence. AI & Society. 2016;31:1–9. doi:10.1007/s00146-015-0638-z.

110. Kuss DJ, Griffiths MD. Social Networking Sites and Addiction: Ten Lessons Learned. International Journal of Environmental Research and Public Health. 2017;14(3). doi:10.3390/ijerph14030311.

111. García-Umaña A. Impacto social y educativo del comportamiento mediático digital contemporáneo: Nomofobia, causas y consecuencias [Social and educational impact of contemporary digital media behavior: Nomophobia, causes and consequences]. Dilemas contemporáneos. 2017;5:1–21.

112. Prasyatiani T, Hijriarahmah D, Solamat LA. Seven Magic Days to Prevent Nomophobia; 2017.

113. Papaconstantinou E, Bartfay WJ, Bartfay E. Smartphone Use, Sleep Quality and Quantity, and Mental Health Outcomes in a University Population. Sleep Medicine. 2017;40:e251. doi:10.1016/j.sleep.2017.11.735.

114. Akun A, Andreani W. Powerfully Tecnologized, Powerlessly Connected: The Psychosemiotics of Nomophobia. In: 10th International Conference on Human System Interactions; 2017. p. 306–310.

115. van Velthoven MH, Powell J, Powell G. Problematic Smartphone Use: Digital Approaches to an Emerging Public Health Problem. Digital Health. 2018;4:2055207618759167. doi:10.1177/2055207618759167.

116. Ǵuzel c. Fear of the age: Nomophobia (No Mobile Phone). Journal of Academic Perspective on Social Studies. 2018;12(20-24).

117. Wang G, Suh A. Disorder or Driver?: The Effects of Nomophobia on Work-Related Outcomes in Organizations; 2018. p. 1–12.

118. Bychkov D, Young S. In: Facing Up to Nomophobia: A Systematic Review of Mobile Phone Apps that Reduce Smartphone Usage; 2018. p. 161–171.

119. Bhattacharya S, Bashar M, Srivastava A, Singh A. NOMOPHOBIA: NO MObile PHone PhoBIA. Journal of Family Medicine and Primary Care. 2019;8:1297. doi:10.4103/jfmpc.jfmpc_71_19.

120. Liu S, Xiao T, Yang L, Loprinzi PD. Exercise as an Alternative Approach for Treating Smartphone Addiction: A Systematic Review and Meta-Analysis of Random Controlled Trials. International Journal of Environmental Research and Public Health. 2019;16(20). doi:10.3390/ijerph16203912.

121. Gezgin D, Hamutoglu N, Sezen-Gultekin G, Yildirim S. Preservice Teachers' Metaphorical Perceptions on Smartphone, No Mobile Phone Phobia (Nomophobia) and Fear of Missing Out (FoMO). 2019;doi:10.14686/buefad.516540.

122. Anshari M, Alas Y, Sulaiman E. Smartphone addictions and nomophobia among youth. Vulnerable Children and Youth Studies. 2019;14:1–6. doi:10.1080/17450128.2019.1614709.

123. Betoncu O, Ozdamli F. The Disease of 21st Century: Digital Disease. TEM Journal. 2019;8(2):598–603.

124. Park CS, Kaye BK. Smartphone and Self-Extension: Functionally, Anthropomorphically, and Ontologically Extending Self via the Smartphone. Mobile Media & Communication. 2019;7(2):215–231. doi:10.1177/2050157918808327.

125. King ALS, Valença AM, Nardi AE. Nomophobia: the Mobile Phone in Panic Disorder with Agoraphobia: Reducing Phobias or Worsening of Dependence? Cognitive and Behavioral Neurology. 2010;23(1):52–4. doi:0.1097/WNN.0b013e3181b7eabc.

126. Yildirim C. Exploring the Dimensions of Nomophobia: Developing and Validating a Questionnaire Using Mixed Methods research. Iowa State University; 2014.

127. Yildirim C, Correia AP. Exploring the Dimensions of Nomophobia: Development and Validation of a Self-Reported Questionnaire. Computers in Human Behavior. 2015;49:130–137. doi:10.1016/j.chb.2015.02.059.

128. Yildirim C, Correia AP. Understanding Nomophobia: A Modern Age Phobia Among College Students. In: Learning and Collaboration Technologies. Springer; 2015. p. 724–735.

129. Yildirim C, Sumuer E, Adnan M, Yildirim S. A Growing Fear: Prevalence of Nomophobia Among Turkish College Students. Information Development. 2015;32(5):1322–1331. doi:10.1177/0266666915599025.

130. Ramos-Soler I, López-Sánchez C, Quiles-Soler MC. Adaptación y Validación de la Escala de Nomofobia de Yildirim y Correia en Estudiantes Españoles de la Educación Secundaria Obligatoria. Salud y Drogas. 2017;17(2):201. doi:10.21134/haaj.v17i2.332.

131. Wahyuningtyas TA, Wibowo ME, Mulawarman M. Metaphor Reality Group Counseling Techniques to Increase Self-Esteem Students with Nomophobia Symptoms. Jurnal Bimbingan Konseling. 2020;9(1 SE).

132. Gutiérrez-Puertas L, Hernández VV, São-Romão-Preto L, Granados-Gámez G, Gutiérrez-Puertas V, Aguilera-Manrique G. Comparative Study of Nomophobia Among Spanish and Portuguese University Students. Nomophobia in Nursing Students. Nurse Education in Practice. 2019;34. doi:10.1016/j.nepr.2018.11.010.

133. González-Cabrera J, León-Mejía A, Calvete E, Pérez-Sancho C. Adaptación al español del Cuestionario Nomophobia Questionnaire (NMP-Q) en una Muestra de Adolescentes. Actas Españolas de Psiquiatría. 2017;45(4):137–144.

134. León-Mejía A, Calvete E, Patino-Alonso C, Machimbarrena JM, González-Cabrera J. Nomophobia Questionnaire (NMP-Q): Factorial Structure and Cut-Off Points for the Spanish Version. Adicciones. 2020; p. 1316.
