# Supplementary material for: A systematic review on nomophobia prevalence: Surfacing results and standard guidelines for future research
Source: PLoS One. 2021 May 18;16(5):e0250509. doi: 10.1371/journal.pone.0250509 (PMC8130950; doi:10.1371/journal.pone.0250509)
Supplement: S1 Checklist — (DOC) [file pone.0250509.s002.doc]

| **Section/topic** | **#** | **Checklist item** | **Reported on page #** |
| --- | --- | --- | --- |
| **TITLE** | | |  |
| Title | 1 | Identify the report as a systematic review, meta-analysis, or both. | We reported in the title that the paper is a systematic review (p. 1) |
| **ABSTRACT** | | |  |
| Structured summary | 2 | Provide a structured summary including, as applicable: background; objectives; data sources; study eligibility criteria, participants, and interventions; study appraisal and synthesis methods; results; limitations; conclusions and implications of key findings; systematic review registration number. | We followed tan IMRAD structure for the abstract presented in page 1, highlighting background, goals, data sources, results, limitations and implications.  We did not register our SR. |
| **INTRODUCTION** | | |  |
| Rationale | 3 | Describe the rationale for the review in the context of what is already known. | This done in pages 1-2. |
| Objectives | 4 | Provide an explicit statement of questions being addressed with reference to participants, interventions, comparisons, outcomes, and study design (PICOS). | The PICO/PECO format (Patient/Problem/Population; Intervention/Exposure; Comparison and Outcomes) is not applicable in our study since the study of nomophobia do not involved clinical or experimental studies with these characteristics.  We address research questions in page 2. |
| **METHODS** | | |  |
| Protocol and registration | 5 | Indicate if a review protocol exists, if and where it can be accessed (e.g., Web address), and, if available, provide registration information including registration number. | This is not applicable in our study since we did not register our SR. |
| Eligibility criteria | 6 | Specify study characteristics (e.g., PICOS, length of follow-up) and report characteristics (e.g., years considered, language, publication status) used as criteria for eligibility, giving rationale. | As said above, The PICO/PECO format (Patient/Problem/Population; Intervention/Exposure; Comparison and Outcomes) is not applicable in our study.  However, we do address years considered, language of the study and eligibility criteria in pages 2 (last paragraph) and 3 (first paragraph). |
| Information sources | 7 | Describe all information sources (e.g., databases with dates of coverage, contact with study authors to identify additional studies) in the search and date last searched. | This is explained in detail in page 3, first paragraph. |
| Search | 8 | Present full electronic search strategy for at least one database, including any limits used, such that it could be repeated. | This is explained in detail in page 3, section 2.2, first paragraph. |
| Study selection | 9 | State the process for selecting studies (i.e., screening, eligibility, included in systematic review, and, if applicable, included in the meta-analysis). | This is done in detail in page 3, first paragraph. |
| Data collection process | 10 | Describe method of data extraction from reports (e.g., piloted forms, independently, in duplicate) and any processes for obtaining and confirming data from investigators. | We did not conduct a meta-analysis.  In page 3, we explained how we removed duplicate studies, and in page 4, section 3, we explained data extraction and present qualitative analysis. |
| Data items | 11 | List and define all variables for which data were sought (e.g., PICOS, funding sources) and any assumptions and simplifications made. | The PICO/PECO format (Patient/Problem/Population; Intervention/Exposure; Comparison and Outcomes) is not applicable in our study since the study of nomophobia do not involved clinical studies with these characteristics.  In page 2, methods, we present our research questions that guided the qualitative analysis. |
| Risk of bias in individual studies | 12 | Describe methods used for assessing risk of bias of individual studies (including specification of whether this was done at the study or outcome level), and how this information is to be used in any data synthesis. | This is explained in page 3, section 2.2: lines 5-10 |
| Summary measures | 13 | State the principal summary measures (e.g., risk ratio, difference in means). | Not applicable in our SR |
| Synthesis of results | 14 | Describe the methods of handling data and combining results of studies, if done, including measures of consistency (e.g., I^2^) for each meta-analysis. | Not applicable in our SR |

Page 1 of 2

| **Section/topic** | **#** | **Checklist item** | **Reported on page #** |
| --- | --- | --- | --- |
| Risk of bias across studies | 15 | Specify any assessment of risk of bias that may affect the cumulative evidence (e.g., publication bias, selective reporting within studies). | Instead of risk of bias the problem that we found and that we focus on is methodological disparity across studies. |
| Additional analyses | 16 | Describe methods of additional analyses (e.g., sensitivity or subgroup analyses, meta-regression), if done, indicating which were pre-specified. | Not applicable in our SR |
| **RESULTS** | | |  |
| Study selection | 17 | Give numbers of studies screened, assessed for eligibility, and included in the review, with reasons for exclusions at each stage, ideally with a flow diagram. | This is explained in page 2 (2.1. Protocol and eligibility criteria), page 3 (first paragraph), and page 4 (PRISMA figure) |
| Study characteristics | 18 | For each study, present characteristics for which data were extracted (e.g., study size, PICOS, follow-up period) and provide the citations. | Not applicable in our SR (we don’t review clinical or experimental studies) |
| Risk of bias within studies | 19 | Present data on risk of bias of each study and, if available, any outcome level assessment (see item 12). | Instead of risk of bias the problem that we found (and that we focus on) is methodological disparity across studies. |
| Results of individual studies | 20 | For all outcomes considered (benefits or harms), present, for each study: (a) simple summary data for each intervention group (b) effect estimates and confidence intervals, ideally with a forest plot. | Not applicable in our SR (we don’t review clinical or experimental studies) |
| Synthesis of results | 21 | Present results of each meta-analysis done, including confidence intervals and measures of consistency. | In the article there are 10 tables and 1 figure showing our analysis of the results extracted from the 108 papers reviewed |
| Risk of bias across studies | 22 | Present results of any assessment of risk of bias across studies (see Item 15). | Not applicable in our SR |
| Additional analysis | 23 | Give results of additional analyses, if done (e.g., sensitivity or subgroup analyses, meta-regression [see Item 16]). | Apart from the 10 tables and figure mentioned above, and after reviewing the 108 papers, we propose standard guidelines for future research in figures 3,3 and 5. |
| **DISCUSSION** | | |  |
| Summary of evidence | 24 | Summarize the main findings including the strength of evidence for each main outcome; consider their relevance to key groups (e.g., healthcare providers, users, and policy makers). | We summarize our main findings in pages 13 to 16 |
| Limitations | 25 | Discuss limitations at study and outcome level (e.g., risk of bias), and at review-level (e.g., incomplete retrieval of identified research, reporting bias). | We discuss limitations in pages 16 to 17 |
| Conclusions | 26 | Provide a general interpretation of the results in the context of other evidence, and implications for future research. | This is done in pages 16 to 17 |
| **FUNDING** | | |  |
| Funding | 27 | Describe sources of funding for the systematic review and other support (e.g., supply of data); role of funders for the systematic review. | This SR did not receive any aid. One of the authors did receive financial to support his research group but not for any specific work, including this one. |

*From:*  Moher D, Liberati A, Tetzlaff J, Altman DG, The PRISMA Group (2009). Preferred Reporting Items for Systematic Reviews and Meta-Analyses: The PRISMA Statement. PLoS Med 6(7): e1000097. doi:10.1371/journal.pmed1000097

For more information, visit: **www.prisma-statement.org**.

Page 2 of 2
